# Jung’s Theory of Dreaming and the Findings of Empirical and Clinical Dream Research

**DOI:** 10.1111/1468-5922.70011

**Published:** 2025-09-06

**Authors:** Christian Roesler

**Affiliations:** ^1^ Freiburg Germany

**Keywords:** analytical psychology, dreams, Jung, psychoanalysis, psychologie analytique, rêves, Jung, psychanalyse, Analytische Psychologie, Träume, Jung, Psychoanalyse, psicologia analitica, sogni, Jung, psicoanalisi, аналитическая психология, сновидения, Юнг, психоанализ, psicología analítica, sueños, Jung, psicoanálisis, 分析心理学, 梦, 荣格, 精神分析

## Abstract

Dreams have been used in psychotherapy since the early days of psychoanalysis, and the effectiveness of therapeutic work with dreams is now well documented. However, there is still no empirically based model for contemporary therapeutic dream work that integrates the findings of empirical and clinical dream research. Structural Dream Analysis (SDA) developed for this purpose is summarized with its research methodology and the results to date. The central assumption is that the agency of the dream ego (the figure in the dream that the dreamer experiences as the ego) to cope with and solve problems in the dream—as opposed to feeling threatened, being anxious and passive and having no solution—can be equated with ego strength in the psychodynamic sense, and that the improvement in the course of therapy is reflected in an increase in dream ego agency. A typology of six dream patterns has been developed that can be used to identify over 90% of dreams in clinical practice. The dream patterns are related to the patient’s specific problems, the themes of psychotherapy and progress in therapy in terms of improvement. The model has been confirmed in a number of empirical studies. The results support Jung’s theory of the dream as a self‐representation of the psyche and his concept of interpretation at the subjective level.

## Abstract (fr)

Les rêves ont été utilisés en psychothérapie depuis les premiers temps de la psychanalyse, et l'efficacité du travail thérapeutique avec les rêves est désormais bien documentée. Cependant, il n'existe toujours pas de modèle empirique pour le travail thérapeutique contemporain concernant les rêves qui puisse intégrer les résultats de la recherche empirique et clinique sur les rêves. L'analyse structurale des rêves (ASD) développée à cet effet est résumée avec sa méthodologie de recherche et les résultats obtenus jusqu'à présent. L'hypothèse centrale est que l'agence de l'ego du rêve (la figure dans le rêve que le rêveur perçoit comme étant l'ego) pour faire face et résoudre les problèmes dans le rêve—par opposition à se sentir menacé, anxieux et passif et ne possédant aucune solution—peut être assimilée à la force de l'ego au sens psychodynamique, et que l'amélioration au cours de la thérapie est reflétée dans une augmentation de l'agence de l'ego du rêve. Une typologie de six schémas de rêve a été développée, qui peut être utilisée pour identifier plus de 90 % des rêves dans la pratique clinique. Ces schémas sont liés aux problèmes spécifiques du patient, aux thèmes de la psychothérapie et aux progrès de la thérapie en termes d'amélioration. Le modèle a été soutenu dans un certain nombre d'études empiriques. Ces résultats soutiennent la théorie de Jung au sujet des rêves en tant que représentations du soi dans la psyché ainsi que son concept d'interprétation au niveau subjectif.

## Abstract (de)

Träume werden seit den Anfängen der Psychoanalyse in der Psychotherapie eingesetzt. Die Wirksamkeit der therapeutischen Arbeit mit Träumen ist inzwischen gut dokumentiert. Dennoch fehlt bis heute ein empirisch fundiertes Modell für die zeitgenössische therapeutische Traumarbeit, das die Erkenntnisse der empirischen und klinischen Traumforschung integriert. Die zu diesem Zweck entwickelte Strukturelle Traumanalyse (STA) wird mit ihrer Forschungsmethodik und den bisherigen Ergebnissen zusammengefaßt. Die zentrale Annahme ist, daß die Handlungsfähigkeit des Traum‐Ichs (der Figur im Traum, die der Träumer als Ich erlebt), Probleme im Traum zu bewältigen und zu lösen – im Gegensatz zu dem Gefühl, bedroht, ängstlich und passiv zu sein und keine Lösung zu haben – mit Ich‐Stärke im psychodynamischen Sinne gleichgesetzt werden kann und daß sich Verbesserung im Verlauf der Therapie in einer Zunahme der Traum‐Ich‐Handlungskraft widerspiegelt. Es wurde eine Typologie von sechs Traummustern entwickelt, mit der über 90 % der Träume in der klinischen Praxis identifiziert werden können. Die Traummuster stehen im Zusammenhang mit den spezifischen Problemen des Patienten, den Themen der Psychotherapie und dem Therapieverlauf im Sinne einer Besserung. Das Modell wurde in zahlreichen empirischen Studien bestätigt. Die Ergebnisse stützen Jungs Theorie des Traums als Selbstdarstellung der Psyche und sein Konzept der Deutung auf subjektiver Ebene.

## Abstract (it)

I sogni vengono utilizzati in psicoterapia fin dall'inizio della psicoanalisi e l'efficacia del lavoro terapeutico con i sogni è ampiamente documentata. Eppure, non vi è ancora un modello empiricamente fondato, per il lavoro terapeutico con i sogni, che possa integrare le scoperte della ricerca empirica con i dati della ricerca clinica sui sogni. L'Analisi Strutturale dei Sogni (Structural Dream Analysis ‐SDA) è stata sviluppata proprio con questo scopo e viene qui sinteticamente presentata con la sua metodologia di ricerca e i risultati fino ad oggi. L'assunzione fondamentale è che la capacità dell'Io del sogno (la figura che il sognatore vive come "io") di affrontare e risolvere i problemi che si presentano, piuttosto che esserne minacciato, angosciato, passivo e non vedere soluzioni, possa essere considerata una misura della forza dell'Io, in senso psicodinamico, e il miglioramento che ha luogo nel corso della terapia riflette l'aumento della capacità di azione dell'Io. È stata sviluppata una griglia di sei pattern che può essere utilizzata per identificare la tipologia per oltre il 90% dei sogni raccolti nel lavoro clinico. I pattern dei sogni sono collegati a specifiche problematiche del paziente, ai temi della psicoterapia ed ai progressi visti come miglioramenti. Questo modello ha trovato riscontro in diversi studi empirici. I risultati confermano la teoria junghiana del sogno che vede in questo una autorappresentazione della psiche e considera l'interpretazione al livello dell'esperienza soggettiva.

## Abstract (ru)

Сновидения используются в психотерапии с первых дней существования психоанализа, и в настоящее время имеется множество подтверждений эффективности терапевтической работы со сновидениями. Однако до сих пор не предложено эмпирически обоснованной современной модели такой работы, которая объединяла бы результаты эмпирических и клинических исследований сновидений. В статье приводится краткое описание разработанного с этой целью метода структурного анализа сновидений (САС), его методологии исследования и результатов, полученных на сегодняшний день. Основная предпосылка заключается в том, что способность эго сновидения (фигуры сновидения, которую сновидец воспринимает как эго) встречаться и справляться с возникающими в сновидении проблемами, не поддаваясь чувству опасности, тревоги, пассивности и беспомощности, может быть связана с силой эго в психодинамическом смысле, а прогресс в терапии проявляется в более активной позиции этого сновидческого эго. Разработана типология, включающая шесть паттернов сновидений и применимая в клинической практике для идентификации более 90% сновидений. Паттерны обусловлены конкретными проблемами пациентов, темами психотерапии и прогрессом в терапии в плане улучшения состояния. Модель прошла клинические испытания в ряде эмпирических исследований. Полученные результаты подтверждают теорию Юнга о сновидении как о самопрезентации психики и его концепцию интерпретации сновидений на субъективном уровне.

## Abstract (es)

Los sueños se han utilizado en psicoterapia desde los primeros tiempos del psicoanálisis, y la eficacia del trabajo terapéutico con sueños se encuentra actualmente bien documentada. Sin embargo, todavía no existe un modelo de base empírica para el trabajo terapéutico contemporáneo con los sueños que integre los hallazgos clínicos y empíricos de la investigación con los sueños. Se ofrece un resumen del Análisis Estructural de los Sueños (AES) desarrollado con este propósito junto con su metodología de investigación y los resultados obtenidos hasta la fecha. El supuesto central es que la agencia del ego onírico (la figura en el sueño que el soñador experimenta como el ego) para hacer frente y resolver problemas en el sueño ‐en oposición a sentirse amenazado, estar ansioso y pasivo y no tener solución‐ puede equipararse con la fortaleza egoica en el sentido psicodinámico, y que la mejora en el curso de la terapia se refleja en un incremento de la agencia del ego onírico. Se ha desarrollado una tipología de seis patrones oníricos que puede utilizarse para identificar más del 90% de los sueños en la práctica clínica. Los patrones oníricos están relacionados con los problemas específicos del paciente, los temas de la psicoterapia y el progreso en la terapia en términos de mejoría. El modelo ha sido confirmado en varios estudios empíricos. Los resultados sustentan la teoría de Jung del sueño como autorrepresentación de la psique y su concepto de interpretación a nivel subjetivo.

## Abstract (zh)

荣格梦理论以及实证和临床方面梦研究的发现

自精神分析早期以来, 梦就被用于心理治疗, 至今, 已有充分文献证明在心理治疗中运用梦是有效的。然而, 目前仍缺乏一个以实证为基础的、能将实证与临床梦研究的最新发现整合起来的, 当代的治疗性梦的工作模型。本文将简介一种为此目标而发展出来的“结构性梦的分析”(SDA), 及其研究方法与迄今所得研究结果。其核心假设是:梦中自我 (梦者体验为“我”的那个形象)的能动性, 即那个在梦里应对与解决问题的能力——而非感到受威胁、焦虑、被动且无解决之道的部分——可与心理动力学意义上的“自我力量”的概念相等同；治疗过程中梦中自我能动性的提高, 可反映治疗的进展。研究者已构建出六种梦的类型, 可涵盖临床实践中 90% 以上的梦境。这些梦的类型与患者的特定问题、治疗主题及治疗进展, 即改善程度相关联。该模型已在多项实证研究中得到验证。研究结果支持荣格关于梦是“心灵的自我表征”的理论, 以及他提出的主体水平释梦的概念。

In psychoanalysis, there has been an ongoing controversy around the understanding of the meaning and function of dreams and the proper approach to dream interpretation that resulted in a plurality of different contemporary approaches (for an overview see Roesler, 2023). Jung, very early, presented a viewpoint different from Freud’s, in which he saw the dream as a symbolic representation of the current situation of the psyche, including unconscious aspects. Following the discovery of REM‐sleep (Aserinsky & Kleitman, 1953), an extensive body of findings has accumulated in the context of empirical dream research. This has influenced the development of psychoanalytic dream theories, and has, by and large—and in contrast to the original intent of this research to falsify psychoanalytic ideas—provided evidence for most of the psychoanalytic theories about dreams. Specifically, dreams carry meaning connected with the waking life of the dreamer and its emotionally relevant topics, and are therefore a helpful tool in psychotherapy, though most of Freud’s original ideas (e.g., that the dream is the protector of sleep) are now outdated (Roesler, 2023). In parallel to empirical dream research, a tradition of clinical dream research has developed conducted mainly by psychoanalytic researchers (Fonagy et al., 2012), which aims at investigating the relationship between dreams, psychopathology and psychotherapy process. Both research traditions can contribute to a clarification concerning the debates in psychoanalytic theories of the meaning of the dream.

An overview of the findings of these research traditions also makes clear that there is still no systematically tested theoretical model of the function and meaning of dreams as they relate to the aims and course of psychotherapy. On this background, the present paper will summarize the findings of the ongoing research project Structural Dream Analysis (SDA), which builds on clinical dream research and enables the formulation of an empirically supported theoretical model of how the content and structure of dreams may be related to the personality structure and psychopathology of the patient, and how changes in a series of dreams over the course of psychotherapy may be related to therapeutic improvement.

## Psychoanalytic Dream Theories

For Freud (1900), the dream is the “guardian of sleep”. As repressed drive excitations threatening for the ego are relegated to sleep rather than waking consciousness, they are transformed in the course of a censorship by the *dream work* (this involves a series of mechanisms, such as condensation, displacement, symbolization, etc.) into dream content, which is no longer threatening for the waking ego. The dream is a wish‐fulfilment. For dream interpretation, this means that starting from the *manifest* dream content, a way must be found back to the *latent* dream content, which Freud emphasizes is only possible through the dreamer’s associations. In this sense, the dream is “the royal road to the unconscious”.

Even in the early psychoanalytic community, this view was not unquestioned (Roesler, 2023). A contrasting view was taken by Jung (1969), where he describes the dream as a “spontaneous self‐portrayal, in symbolic form, of the actual situation in the unconscious” (p. 263). In his view, the manifest content is not distorted by dream censorship but is exactly what it portrays. Jung developed the interpretation on the subjective level, which means all the figures appearing in the dream are considered as personifications of components of the dreamer’s personality; this allows the dreamer’s hitherto unconscious components to be made conscious. Accordingly, the dream has a compensatory function in relation to consciousness, and so offers possible solutions to conflicts, or at least makes suggestions as to how the problematic situation can be considered from a broadened perspective. It is therefore attributed a self‐healing as well as a creative potential.

Although Jung himself repeatedly stated that he had no dream theory, two different dream theories can be identified in his work:

1. the dream is a spontaneous self‐representation of the situation of the psyche.
2. the dream compensates for the conscious attitude.

In practical dream work, the dream is therefore always considered in terms of what its compensatory aspect is in relation to the current attitude of the conscious mind (Jung, 1944). In Jung’s view, the symbols and images in dreams are not distortions of the actual dream content, but correspond to the usual language of the unconscious. Some of these symbols may have archetypal character, which means—as Jung assumes—they convey universal meanings which are not restricted to the individual context of the dream or the dreamer.

## What Empirical Dream Research can Contribute to Understanding the Dream

In summarizing the results of empirical dream research, Barrett & McNamara (2007) suggest that in the dreaming state, the brain is in a mode where it does not have to process new input but can use larger capacities for working on already perceived problems and finding creative solutions. The dreaming mind especially focuses on experiences in waking life that have emotional meaning for the dreamer, and it can find solutions for problems more readily compared to waking consciousness because it is able to interconnect different areas and functions of the brain (Hartmann, 2010). In contemporary theories of dreaming there is general agreement that dreams reflect the efforts of an individual to adapt to reality: the dream is “an unconscious attempt to find a solution to an emotionally relevant concern. In dreams people think about their main concerns, particularly those concerns that they have been unable to solve by conscious thought alone, and try to develop and test plans and policies for dealing with them” (Gazzillo et al., 2020, p. 187). They further suggest that the models of dream functions and meanings based on empirical dream research “assume that (a) dreams are orderly and not random experiences; (b) dreams are meaningful, that is, the content of dreams is overtly or covertly related to the waking life of the dreamers; and (c) dreams play some important psychological function” (Gazzillo et al., 2020, p. 187).

In general, research on the content of dreams found that it is closely and systematically correlated to various dimensions of the waking life of the dreamer (Zadra & Domhoff, 2010; Iftikhar et al., 2020), this being the reason why the most prominent theory in dream research today is the so‐called *continuity hypothesis* (Schredl et al., 2022). It states that there is no fundamental difference between waking state and dream mentation, which counters Freud’s theory of a censorship and resulting distortion of the latent dream material into the manifest dream (see Roesler, 2023). Object relations in dreams, such as feeling controlled and oppressed by the mother, were strong predictors of therapist’s judgements of their patient’s levels of object relations in waking life (Eudell‐Simons et al., 2005).

Hall and Van de Castle (1966), who developed a coding system for the content of dreams, argued that it is possible to draw a personality profile based exclusively on the person’s dreams. Furthermore, while there is substantial continuity in the personal dream themes over a long period of time (Levin, 1990), the themes change when a person goes through psychotherapy (Cartwright, 1977). Greenberg & Pearlman (1978) go so far beyond Freud in the interpretation of their results as to say that it is enough to know the conflictual themes in the dreamer’s waking life to translate the content of his dreams—the quest for an encoded latent meaning content becomes superfluous (for a more detailed discussion, see Roesler, 2023).

Extensive empirical studies have also been carried out by the psychoanalysts Glucksmann & Kramer (2015). A very clear connection could be made between the dreamer’s emotionally significant experiences and the themes that arose in the dreams. In sleep laboratory studies, they were also able to show that in one night’s dreams, one prominent emotional theme was repeatedly reworked, and that this dream content did not fundamentally change even over a 20‐night period. They explicitly proposed that the emotional intensity of the experience determines its effect on the dream. This effect is so strong that independent evaluators could identify the person’s immediate and long‐term significant themes from dreams alone.

## Clinical Dream Research

Independently of empirical dream research, there is a research tradition in psychoanalysis investigating dreams and their significance for psychotherapy (Fonagy et al., 2012), starting with Alexander (1925), who was also the first to systematically investigate dream series in addition to single dreams. In contrast to the empirical research presented above, which investigates the connection between dreams and waking life in general, clinical dream research aims at investigating the relations between dreams and psychopathology, including therapeutic change over the course of therapy. This research aimed at overcoming the solely interpretive approach usually applied in psychoanalytic practice, which was criticised very early on by psychoanalysts themselves. For instance, the Jungian author K. W. Bash (1988/1950) wrote: “It certainly seems to me that a considerable and not unfounded part of the doubt about dream analysis as science derives from the fact that it almost invariably begins with an interpretation of content that allows great subjective scope with relatively limited possibilities of objective verification” (p. 145, translated quotation). More recent psychoanalytic research papers on dreams generally examine connected dream series over the course of the psychotherapy since it was found that logical structures of various dreams in the same person are interconnected so that all dreams serve as components of a single communication structure (Fischmann et al., 2012)—a viewpoint which was already put forward by Jung (1944).

Not only do dreams provide information about a patient’s functioning in their waking life, but there is further evidence that dream content can reflect changes or improvement in a patient throughout the psychotherapeutic process. A number of case studies (Eudell‐Simons et al., 2005) found significant changes in dream content over the course of therapy, e.g., that changes in dreams reflected the clinical improvement by more adaptive and integrated actions of dealing with problems and conflicts. The overall pattern of patients’ dreams throughout the therapeutic process corresponded to progressive changes in personality structure, self‐representation, defensive configurations, interpersonal relationships, transference reactions, and resolution of core conflicts. Patients who improved over the course of psychotherapy compared to non‐improved patients showed significant changes in the manifest dream content in the former, in contrast to only little change in the dream content in the latter (Eudell‐Simons et al., 2005).

Ellis (2016) investigated qualitative changes in nightmares from five patients suffering from PTSD after successful trauma‐oriented treatment. On completing treatment, the dream ego actions progressed from passive to active responses, e.g., shifting from freeze to flight or fight, finding their voices, seeking help or acting.

A series of prominent investigations within German‐speaking psychoanalytic dream research is based on records of psychoanalytic treatments in the context of the Ulm Textbank (overview in Fischmann et al., 2012). Leuzinger‐Bohleber (1989) examined 112 dreams from five long‐term psychoanalytic therapies, consisting in each case of dreams from the first and last 100 sessions. She ascertained that in successful treatment, the dreams at the end of the therapy differed from those at the beginning, which was not the case in unsuccessful therapy. In successful therapy, the spectrum of affects in the manifest dream content expanded, and anxiety dreams were less frequent than at the beginning of the treatment. The patients’ capacity for successful problem‐solving increased, and the dream ego was more active and less frequently in the observer position. Leuzinger‐Bohleber (2013) states that “the problem solutions in the dream contain references to turning points in the psychoanalytic treatment and therapeutic changes” (p. 267, translated quote).

The German study on long‐term treatment of chronic depression (Fischmann & Leuzinger‐Bohleber, 2018) in which dreams were investigated, similarly found that in successful psychoanalytic therapies there are positive changes of the dream atmosphere, more successful problem‐solving in the dreams; the dream ego changes from a perspective of an observer to active involvement, and there are more helping figures in the dream. Döll‐Hentschker (2008) used the coding model of Moser & von Zeppelin (1996) to examine 142 dreams from five psychoanalyses and obtained very similar results: intra‐individual differences between the beginning and end of treatment when there was a positive outcome, as opposed to minor or negative changes in failed treatments. Similar results were also found by Kächele in various studies (overview in Kächele, 2012). Kächele concludes that changes during the treatment are clearly reflected in the change in the structure of the dreams (see also Glucksman & Kramer 2004, 2011, 2012).

It is now well established that working with dreams in psychotherapy contributes significantly to improvement over the course of therapy, compared to therapies in which dreams were not addressed (Fiss, 1979; Hill, 1996, 2004; Glucksman & Kramer, 2011). Eudell‐Simmons & Hilsenroth (2005) identify “four contributions of dreams to psychotherapy … as being particularly relevant for clinicians in applied practice to (I) facilitate the therapeutic process, (II) facilitate patient insight and self‐awareness, (III) provide clinically relevant and valuable information to therapists and (IV) provide a measure of therapeutic change” (p. 260).

These findings provide clear insight into the existence of a general connection between dreams and the course of therapy; on the other hand, there is still no research methodology which enables the analysis of the meaning of dreams in the course of therapy.

## The Research Project “Structural Dream Analysis”

Frequently used methods to investigate the meaning of dreams involve the coding of content elements in the dreams, e.g. the occurrence of certain symbols (Hall & Van de Castle, 1966). The problem with this approach is that the elements of the dream are taken out of context, the context serving as a background for both the individual dream and the whole dream series. In psychoanalytic research on dreams there is often the problem that certain assumptions about the function of dreams are taken for granted, e.g., the idea that the function of dreaming is to protect sleep, and so such an approach is unable to falsify any Freudian assumptions.

In the method of Structural Dream Analysis (hereafter SDA, Roesler, 2018a), the inclusion of any theoretical assumptions about the function of dreaming was minimized. SDA assumes that the meaning of dreams is not so much transmitted by the elements or symbols in the dream but by their interrelationships, or “structure”. 1 It is especially important in the relationship of the dream ego to other figures and elements in the dream, and the extent of agency of the dream ego. This approach follows Jung’s idea of interpretation on the subjective level, which focuses on identifying patterns rather than on coding elements, especially whether the dream ego is actively authoring what is happening in the dream or is subjected to other figures’ actions. SDA sees the dream as a narrative, which allows for the use of analytic tools developed in narratology (for more details see Roesler, 2018a). Here, a narrative is defined as a development from a starting point, which constitutes a problem in need of repair or solution, and the protagonists’ ways of dealing with the problem and eventually solving it. Thus, a dream can be seen as a short story about how the protagonist, in most cases the dream ego, addresses a problem. From this perspective, SDA is a qualitative, interpretive research method that attempts to formalize the process of interpretation of the dream in a way that the conclusions are independent from the interpreter. The meaning conveyed by the dream is analysed in a systematic series of interpretive steps for which a formalized manual is available (Roesler, 2018a).

### The Phases of Research with SDA

In the first phase, where SDA methodology was developed, a complex set of (narratological) methods of analysis was applied to well‐documented single cases in an intensive, bottom‐up manner of qualitative analysis. The researchers attempted to identify typical, repetitive patterns present in the dream series of a case from the text alone, without carrying theoretical concepts into the material. A detailed description of the methodology of this first phase of SDA and its application to a case example was published earlier in this journal (Roesler, 2019). In this first phase, 15 well‐documented cases with a total of 206 dreams were thoroughly investigated with this extensive, single case analysis form of SDA (for details about these cases see Roesler, 2018b). In the second phase, with the above 15 cases used as a basis, a cross‐case analysis was conducted, which aimed at identifying typical patterns of dreams between different patients. A qualitative analysis was conducted aiming at identifying categories typical for a number of cases in the sample and formulating a theoretical model. These patterns were correlated with the initial level of psychopathology of the patients as well as with the course of therapy and improvements gained. The crucial explanatory variable was found to be the agency of the dream ego. As a result of this phase 2, a typology of dream patterns was created (see Table 1) as well as a theoretical model of the correlations between dreams/dream patterns, initial psychopathology, and the course of psychotherapy (for details see Roesler, 2018b). With this typology, it was possible to categorize more than 90% of the dreams in the study.

**Table 1:** Typology of dream types/patterns

**Type 1: No dream ego present** The dream ego does not participate in the plot (dreamer observes a scene as if watching a movie)
**Type 2: The dream ego is threatened** The dream ego is threatened or pursued by a threatening figure
**2.1** The dream ego is completely destroyed, damaged, dismembered, severely wounded, or even killed
**2.2** The dream ego is overwhelmed, i.e. is completely powerless, no coping strategy
**2.3** The dream ego flees from the threat
**2.4** The dream ego defends itself, i.e., has a strategy, but the threat remains
**2.5** The dream ego successfully defends itself against the threat (threat transforms to non‐dangerous)
**Type 3: The dream ego is confronted with a performance requirement** The dream ego encounters a performance requirement (e.g., an examination) set by other characters in the dream: find something (which was previously lost), give something to someone, etc.
**3.1** The dream ego fails (e.g., fails a test); is subjected to the control of others, against its will, cannot do anything about it
**3.2** The dream ego is prepared, but encounters obstacles. The task is ultimately not solved
**3.3** The dream ego is subjected to the requirement, but successfully copes with it through its own activity
**Type 4: Mobility dream** The dream ego is on the way somewhere (specified or unclear destination): it follows its own initiative, tries to implement its own intentions or plans
**4.1** The dream ego is locked up in a room, trying to find its way out or to break out, but failing.
**4.2** The dream ego wants to move, e.g., travel, but has no means, e.g., misses the train.
**4.3** The dream ego moves successfully but encounters obstacles and the process cannot be continued.
**4.4** The dream ego is in motion, encounters obstacles, and the desired destination is not reached.
**4.5** The dream ego manages to move successfully and reaches the desired destination.
**Type 5: Social interaction** The dream ego is trying to communicate with someone, to create a satisfying encounter (includes sexuality).
5.1 The dream ego wants to get in contact with others, but is ignored by the others.
5.2 The dream ego comes into contact with others but encounters obstacles; all in all, the attempt to establish a desired contact fails.
5.3 The dream ego is successful in establishing the desired contact.
**Type 6: Autonomy dream** The dream ego establishes or defends its autonomy.
**6.1** The dream ego is flooded by the affection of others.
**6.2** The dream ego is aggressive towards others (even kills them), which expresses the will of the dream ego to be separate and independent from others.
**6.3** The dream ego is on its own and content.
**6.4** The dream ego helps others (has so many resources left that it can provide them to the others, but it is the initiative of the dream ego)

The dream patterns represent the general idea of SDA that the dream forms a micro‐narrative in which a problem is presented that the dream ego has to struggle with, the ego is confronted with a challenge and attempts to fulfil a plan or task. The agency of the dream ego rises continually, starting from Pattern 1 (no ego present at all); to Patterns 2 and 3, where the dream ego is present but under pressure from other forces in the dream, and the initiative is not with the ego but with their power and control; to Patterns 4 and 5, where the ego has taken over the initiative and attempts to follow a personal plan; to Pattern 5, where the focus is on creating satisfying relationships with others; and, finally, to Pattern 6, where the dream ego gains full autonomy from other elements and can operate freely. The same movement towards more autonomous agency of the dream ego applies to each of the patterns, e.g., inside pattern 2 there is a movement from pattern 2.1 (a completely powerless dream ego) to 2.5, where the dream ego successfully overcomes the threat.

### The Theoretical Model

The dream patterns can be interpreted psychologically as an expression of the capacity of the dreamer’s ego, on different levels, to regulate and cope with emotions, motivations and unresolved inner conflicts. The extent of agency of the dream ego is equivalent to what psychoanalysis calls ego strength or maturity of the personality, i.e., the degree of integration of the ego and other parts of the psyche into the whole of the personality and the patient’s conscious decision‐making (Blanck & Blanck, 1974).

**Figure 1:**
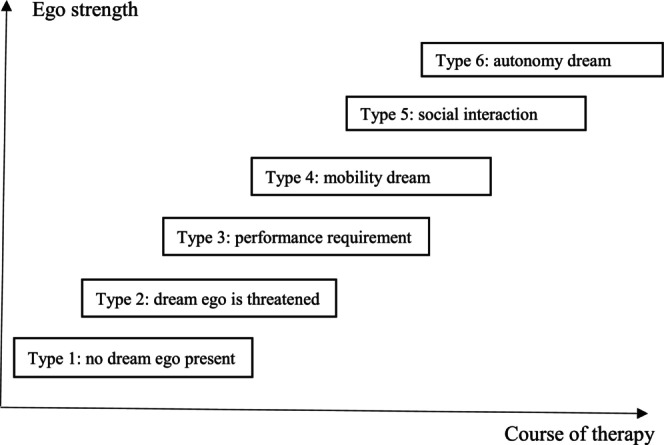
Dream patterns, ego strength and course of therapy

In those cases where psychotherapy was successful, as evidenced by an improvement in symptoms, psychological well‐being, emotional regulation and, from a psychoanalytic point of view, a gain in psychological structure/integration and ego strength, we found a typical pattern of transformation in the structure of the dreams (see Figure 1). Typically, the initial phase of the psychotherapeutic process was dominated by a repetitive pattern in the patient’s dreams, which showed a weak dream ego incapable of solving the problem presented in the dream, such as the dream ego being threatened with no strategy to cope with the threat, fleeing or attempting to hide. In Pattern 3 dreams, where the dream ego has to fulfil a task, it typically fails, is not prepared, acts too late, etc. In mobility dreams (Pattern 4), the dream ego typically fails to reach the desired aim, is on the wrong bus or train, or has no ticket, etc. If psychotherapy is successful, the typical patterns change into more successful activities of the dream ego: it confronts threatening figures, fights actively, and successfully overcomes the threat, successfully fulfils the tasks (e.g., passes an exam) or succeeds in reaching the desired aim.

In general, there is a movement from lower patterns (1, 2 and 3) dominating the first half of the dream series, where the dream ego is subjected to others’ initiative or feels threatened, towards patterns 4, 5 and 6 in the second half of the dream series, where the dream ego gains more and more agency and solves the problem in the dream successfully. For instance, it may be more capable of creating satisfying interactions with others, including sexual encounters, or makes itself more independent from others. This transformation is interpreted from a psychodynamic perspective as speaking to the fact that an initially weak ego structure, which fails to regulate and integrate threatening emotions; or impulses, gains in ego strength over the course of the therapy, and succeeds in coping with initially repressed or split‐off parts of the psyche, integrating them into constructive interactions with others. These gains are reflected in real life by the ego becoming more capable to execute adaptive choices, conduct its plans, reach its aims, and express its needs in social interactions.

In addition to this model of transformation, we also hypothesize that there is a close connection between the initial level of ego strength/structural integration of the patient’s personality at the start of therapy, and the level of dominating pattern in the first half of the dream series, in the sense that the scale of ego strength/personality integration is reflected in the scale of dream patterns. For instance, patients whose dreams are shaped mainly by the threat‐escape pattern usually struggle with structural problems around an unstable ego and personality, whereas patients with dreams of mobility and interpersonal problems seem to have more integrated personalities and higher ego strength, and tend to be preoccupied with more neurotic and interpersonal problems.

### The Central Complex Represented in the Dream

Beyond that, we hypothesize that the relationship between the dream ego and threatening figures, and the reaction of the dream ego to the threat, represent the relationship between actual ego strength and unintegrated or conflicted parts of the psyche, i.e., complexes. The special form the threatening figure takes in the dream—especially if the dream pattern is repetitive—can be seen as symbolizing the psychological problem, the central complex with which the dreamer is struggling.

### Agency of the Dream Ego

There are research findings which support the above hypothesis concerning agency of the dream ego. Varvin et al. (2012) demonstrated that dreams of PTSD‐patients showed destruction of the dream ego, threatening elements, and passive behaviour of the dream ego, in contrast to high agency of the dream ego in the control group with no PTSD‐diagnosis. The author found a relationship between psychopathology of the dreamer, dream structure and agency of the dream ego and concludes that the dream provides information about the dreamer’s ego strength. Ellis (2016) found typical changes in the dreams of PTSD‐patients after they had received treatment, moving towards more successful dream ego agency. Schredl (2018) confirms the idea that in nightmares the failing capability of the dream ego is pictured to deal with inner conflicts and problems.

Sándor, Szakadát and Bódizs (2016) found that the presence and activity of the dream ego in the dreams is strongly correlated with the extent of effective coping and emotion regulation in the waking life of the dreamer. According to Foulkes (1999), in young children’s dreams, there is usually no dream ego, this only emerges at the age of about seven, parallel to the development of ego strength. In a study by Euler et al. (2016) a correlation was found between the level of personality integration in the form of maturity of defence mechanisms and dream imagery.

## Empirical Testing of the Theoretical Model of SDA

After the development of the system of dream patterns (see Table 1), in the last years the research team was occupied with empirically testing the theoretical model of SDA; in the following, the results are presented. First, three hypotheses were extracted from the theoretical model:

In cases therapy is successful, an upward movement through the dream patterns over the course of the dream series can be found, thus a correlation exists between the course of psychotherapy and the course of the dream series’ movement through the different dream patterns.

The agency of the dream ego increases over the course of therapy if the therapy is successful.

The severity of the psychopathology is reflected in the initial level of the dominant dream pattern.

The material for the empirical testing had to meet a number of criteria: the case is completed; detailed diagnostic information must be available about the case as well as about the course and results of therapy, and the case must include a series of dreams which ideally cover the whole course of therapy. Material from different sources was used:

- A sample of 150 case reports from the archives of the C. G Jung‐Institut Stuttgart with varying numbers of dreams per case was examined. These extensive case reports were compiled by training candidates about their training cases (compulsory part of the training requirements) in the years 1991–2018. They contain detailed diagnostic information (ICD diagnosis, biographical data, psychopathological symptoms, a psychodynamic model of the personality, major complexes, etc.), as well as a detailed account of the course of therapy, the major themes that were worked on, an evaluation of the development of the personality, the improvements gained, and the overall results of therapy.
- Famous cases from the history of psychoanalytic psychotherapy research that were published in the literature and contained a full series of dream reports.
- We specifically looked for counterexamples to our hypotheses, e.g., failed cases, dream series from persons who never attended psychotherapy, etc., for which the theoretical model would predict no transformation/upward movement in the dream patterns.

For the investigation of the dream series, the typology of dream patterns (Table 1) was used as a coding system and values were assigned to the categories (e.g., dream pattern 1 = 1, dream pattern 2.1 = 2, dream pattern 2.2 = 3, etc.). In the same manner, values were assigned to the different levels of dream ego agency. Each series of dreams was coded by at least two independent raters, who received training based on the above dream manual. Inter‐rater reliability was assessed and found to be good. For evaluating the initial severity of the psychopathology of the patients in reference to the level of personality structure/structural integration, a rating scale was developed for use in the research project based on the levels of personality structure described in Operationalized Psychodynamic Diagnostics (OPD Task Force, 2008).

Details of the statistical analyses and the results are published in Roesler et al. (2024). The results clearly support the three hypotheses investigated. In those cases in which the therapists considered the therapy successful, a clear upward movement of the dreams through the dream patterns could be observed, with a parallel significant rise in dream ego agency. In contrast, no such development could be observed in a dream series (containing 208 dreams covering a period from 2017 to 2020) provided by a person who had never received psychotherapy 3.

One case was reported as a so‐called negative therapeutic reaction, the patient feeling rejected, showing strong resistance, and finally retreating into silence. Around session 25 the therapist identified evidence of destabilization, paranoid anxieties and depersonalization in the patient, and the therapy was terminated. In the report, the therapist regarded the case as failed. Statistical analysis of the coded dream series found that the curve of dream patterns showed a downward movement. Interestingly, the collapse of the therapeutic relationship around session 25 can be identified in the dream patterns as the point of strong regression to lower dream patterns representing a strong reduction in ego strength.

Regarding the third hypotheses, a clear connection was found between initial level of psychopathology and dominant dream patterns at initiation of therapy. The majority of the patients with low structural integration of the personality showed an accumulation of dream patterns 1 or 2 in the first phase of therapy compared to patients with higher structural integration. The majority of higher structured personalities (moderate or good structural integration of the personality) dreamed predominantly in the higher dream patterns. Those patients with the highest structured personalities (neurotic conflict) rarely showed patterns 1 or 2 in the first half of the therapy.

These findings support the theoretical model which was outlined above. Additionally, several in‐depth single case studies confirmed this, and will be presented in the following.

## Single Case In‐depth Studies

### Amalia X

The so‐called specimen case Amalia X is a Freudian psychoanalysis of more than 500 sessions, which was fully documented on video and transcribed in the context of the Ulmer Textbank, and was the subject of a number of empirical investigations (Kächele, 2012; Kächele, Eberhardt & Leuzinger‐Bohleber, 1999; Kächele et al., 2006). It is considered to be the best investigated case in the history of psychotherapy research. This case included 95 dreams which were discussed over the course of therapy. These dreams were subject of a number of studies as well (for an overview see Roesler & Widmer, 2023). The following information on the case is taken from Kächele et al. (2006): The female patient (35 years of age at the beginning of therapy) suffered from a disorder of her self‐esteem and recurring episodes of depression. The deficits in her self‐esteem were related to her hirsutism—the virile growth of hair all over her body since puberty, which resulted in social insecurity. Her defence mechanisms included compulsion neurosis as well as symptoms of anxiety, and erythrophobia (fear of blushing). These problems had a negative effect on her ability to form personal relationships and even more so sexual relationships—at the beginning of therapy she had not had any sexual contacts.

A number of standardized measures were applied to document the success of therapy. Results of the treatment show significant improvement, a reduction in psychosomatic symptoms, stabilization of mood and self‐esteem, and a rise in extraversion. These results remained stable in the two‐year follow‐up (Kächele et al., 2006).

In our statistical analysis of the 95 dreams, each of the above hypotheses was confirmed. We found a significant upwards movement from lower to higher patterns as well as a gain in initiative of the dream ego over the course of therapy. There is an accumulation of dream pattern 2 in the first phase of therapy, whereas the second half of therapy is dominated by dream pattern 5. One of the last dreams is a characteristic autonomy dream, in which the dreamer walks on her hairs (note the specific disorder of hirsutism) into the practice of her analyst, says goodbye and leaves on her own.

### The Case C. L.

A well‐known German psychoanalyst, Alexander Mitscherlich (1983) published the well‐documented case C. L. containing 103 dreams, which were discussed over the course of therapy, together with a detailed case description. The case is considered to be highly successful. The 28‐year‐old female patient was treated because of sleep disturbances, depression, anxiety, psychosomatic symptoms (e.g., unexplained fever attacks), and diverse sexual problems (e.g., vaginismus), which were apparently connected to repeated experiences of sexual assault during adolescence.

Again, the statistical analyses confirmed the hypotheses. Parallel to the progress of therapy, there is a movement from lower to higher dream patterns, with the second half of therapy being dominated by patterns 4 and 5. Over the course of therapy, the dream ego increasingly succeeds in coping with tasks, solving problems and creating satisfying social interactions; and there is a continuous increase in dream ego agency.

Based on the dreams alone, a psychological profile of the dreamer was created, which correctly predicted the symptoms and psychological problems described in the case report. The images and topics appearing repeatedly in the dreams provided an accurate picture of the psychological conflicts of the patient as well as of therapeutic change over the course of therapy. For example, a major topic in the dream series is encounters with men, which in the first half of the dream series are generally unpleasant and, in many cases, even threatening. This motif changes over the course of therapy, encounters with men become more satisfying, and by the end of the dream series men even become rescuers of the dream ego in threatening situations (change in the motif from pattern 2 to pattern 5 dreams). In contrast to the case of Amalia X, the beginning of this series contains a number of pattern 1 dreams, which suggest a low level of ego strength. This is confirmed by the case report, arguing for a more severe disorder of self‐esteem in the patient.

### A Short‐Term Therapy Case

Both cases presented above are classic long‐term psychoanalytic psychotherapies. In contrast to that, the three hypotheses tested here were also investigated in a detailed single case analysis of a patient in short‐term psychodynamic psychotherapy providing a series of 26 dreams. Additionally, the patient was tested with a set of standardized clinical and personality measures, by which the initial level of personality structure and psychopathology was evaluated. The single case analysis confirmed all of the three hypotheses (Vömel, 2020).

A new publication on classic cases of psychoanalytic dream interpretation re‐analysed with SDA is under review at the moment and will provide more in‐depth case analyses including Jung’s famous dream cases (Roesler, in review).

## Discussion and Conclusion

The findings of SDA which have accumulated over the last years provide support for central elements of Jung’s theory of dreaming (Jung, 1969). The viewpoint that the dream provides a picture of the current situation of the psyche, and in particular the approach to interpret all elements in the dream as representations of parts of the psyche, are supported quite clearly. Concerning the connection between initial level of psychopathology and dominant dream patterns at initiation of therapy, the results support the hypothesis that the personality structure, and thus psychopathology, of patients is closely related to the dream patterns. In addition to this general support, there are more details found in the research which support Jungian ideas.

Typically, there is a fluctuation in dream types across a dream series instead of a linear progression, which reflects the typical nonlinear improvement in therapy processes—and this is something which Jung (1969) pointed out. As a part of this nonlinear development, typically in the mid‐phase of therapy, a “regression” to a series of pattern 2 dreams can be found, which we interpret as a consequence of developing a secure therapeutic relationship in the initial phase of therapy. The patient’s ego complex is thus strengthened and becomes capable of confronting the central complex (“the path of the hero”), which initially appeared in the imagery of the dream ego being threatened. Following this “confrontation phase”, a quick rise towards patterns 5 and 6 can be observed, which marks the termination phase of therapy.

The dream can be seen as an imaginative space where the inner drama of conflicting forces in the psyche unfolds. The stronger the inner conflicts, the more dramatic are the challenges to the dream ego. In parallel to the support the ego receives in the therapeutic relationship, it becomes more capable to confront the unconscious forces, to cope with them, and finally reaches a level of autonomy in which it can make more autonomous adaptive choices. In the course of this increasing integration, formerly split‐off aspects/impulses, which appeared as threatening figures, change into helpers for the dream ego. In that sense, dream ego agency, as defined in the structural model presented here, is a measure of ego strength and psychological integration of the patient’s personality.

Dreams in psychotherapy mirror the personality and psychological problems as well as the development of the person over the course of psychotherapy. The developing ego strength of the patient is reflected in the scope of action that the dream ego is able to initiate in relation to other subjective figures, as documented by the dream patterns described by SDA. Thus, the information about the personality structure is not just shown in static symbols and images but rather in patterns of the relationship between the dream ego and other figures in the dream.

Based on these findings it could be questioned that the dream has a compensating function in relation to the attitude of consciousness, as Jung (1969) theorizes. The function of dreams could be called not so much compensating, but more confronting consciousness with the actual reality of the psychological situation. It is not possible, given the limited space of this paper, to discuss this question in depth here (see Roesler, forthcoming publication, for an extensive discussion).

We found no support for the assumption that there are typical meanings connected with specific symbols. For example, in one case of a female dreamer, the dream ego was repeatedly threatened by snakes. The therapist diagnosed an unresolved conflict between a highly moralistic superego on the one hand and very lively sexual desires on the other. The snake here can be interpreted as a sexual, phallic symbol, which appears threatening to an ego under the pressure of the moralistic superego. In contrast, in the dreams of a young man, the snake repeatedly acted as a helper. To sum up, symbols appearing repeatedly in a dream series can often be interpreted as symbolic images for parts of the psyche, such as its impulses and complexes which are not yet integrated into the whole of personality, and which therefore appear threatening to ego integrity. But the symbol has to be interpreted in the context of the personality of the dreamer, their life circumstances, and capacity to adapt to them.

## Footnotes

^1^
Not to be mistaken with the psychoanalytic use of the term structure in the sense of structure of personality.

^2^
Initially, there were only five patterns, to which later the sixth pattern (autonomy dreams) was added as a result of the analysis of further material.

^3^
Thanks to Prof. Dr. Michael Schredl, Zentralinstitut für seelische Gesundheit, Mannheim/Germany.

## References

[joap70011-bib-0001] Alexander, F. G. (1925). Über Traumpaare und Traumreihen. Internationale Zeitschrift für Psychoanalyse, 11, 80–85.

[joap70011-bib-0002] Aserinsky, E. , & Kleitman, N. (1953). Regularly occurring periods of eye motility and concomitant phenomena during sleep. Science *,* 118, 273–274. 13089671 10.1126/science.118.3062.273

[joap70011-bib-0003] Barrett, D. , & McNamara, P. (2007). The new science of dreaming: Vol. 2. Content, recall, personality correlates. Praeger.

[joap70011-bib-0004] Bash, K. W. (1988/1950). Zur experimentellen Grundlegung der Jungschen Traumanalyse. In: Bash, K.W. , Die analytische Psychologie im Umfeld der Wissenschaften (S. 145–154). Huber.

[joap70011-bib-0005] Blanck, G. , & Blanck, R. (1974). Ego psychology: Theory and practice. Columbia University Press.

[joap70011-bib-0006] Cartwright, R. D. (1977). Night life. Prentice‐Hall.

[joap70011-bib-0007] Döll‐Hentschker, S. (2008). Die Veränderung von Träumen in psychoanalytischen Behandlungen. Brandes & Apsel.

[joap70011-bib-0008] Ellis, L. A. (2016). Qualitative changes in recurrent PTSD nightmares after focusing‐oriented dreamwork. Dreaming, 26(3), 185.

[joap70011-bib-0009] Eudell‐Simmons, E. M. , & Hilsenroth, M. J. (2005). A review of empirical research supporting four conceptual uses of dreams in psychotherapy. Clinical Psychology and Psychotherapy *,* 12(4), 255–269. 10.1002/cpp.445

[joap70011-bib-0010] Euler, J. , Henkel, M. , Bock, A. , & Benecke, C. (2016). Strukturniveau, Abwehr und Merkmale von Träumen. [Structural level, defence and characteristics of dreams]. Forum der Psychoanalyse *,* 32 *,* 267–284. 10.1007/s00451-016-0243-x

[joap70011-bib-0011] Fischmann, T. , Kächele, H. , & Leuzinger‐Bohleber, M. (2012). Traumforschung in der Psychoanalyse: klinische Studien, Traumserien, extra klinische Forschung im Labor. Psyche *,* 66, 833–861.

[joap70011-bib-0012] Fischmann, T. , & Leuzinger‐Bohleber, M. (2018). Traum und Depression. In Berner, W. , Amelung, G. , Boll‐Klatt, A. , & Lamperter, U. (Hg.), Von Irma zu Amalie. Der Traum und seine pychoanalytische Bedeutung im Wandel der Zeit. Psychosozial.

[joap70011-bib-0013] Fiss, H. (1979). Current dream research. A psychobiological perspective. In Wolman, B. (Ed.), A handbook of dreams. Van Nostrand.

[joap70011-bib-0014] Fonagy, P. , Kächele, H. , Leuzinger‐Bohleber, M. , & Taylor, D. (2012). The significance of dreams: Bridging clinical and extraclinical research in psychoanalysis. Karnac.

[joap70011-bib-0015] Foulkes, D. (1999). Children’s dreaming and the development of consciousness. Harvard Universities Press.

[joap70011-bib-0016] Freud, S. (1900). Die Traumdeutung . *GW* II/III. Fischer.

[joap70011-bib-0017] Gazzillo, F. , Silberschatz, G. , Fimiani, R. , De Luca, E. , & Bush, M. (2020). Dreaming and adaptation: The perspective of control‐mastery theory. Psychoanalytic Psychology, 37(3), 185–198.

[joap70011-bib-0018] Glucksman, M. L. , & Kramer, M. (2004). Using dreams to assess clinical change during treatment. Journal of the American Academy of Psychoanalysis and Dynamic Psychiatry, 32(2), 345–358. 15274500 10.1521/jaap.32.2.345.35276

[joap70011-bib-0019] Glucksman, M. L. , & Kramer, M. (2011). The clinical and predictive value of the initial dream of treatment. Journal of the American Academy of Psychoanalysis and Dynamic Psychiatry, 39(2), 263–283. 21699352 10.1521/jaap.2011.39.2.263

[joap70011-bib-0020] Glucksman, M. L. , & Kramer, M. (2012). Initial and last manifest dream reports of patients in psychodynamic psychotherapy and combined psychotherapy / pharmacotherapy. Psychodynamic Psychiatry, 40(4), 617–634. 23216399 10.1521/pdps.2012.40.4.617

[joap70011-bib-0021] Glucksman, M. L. , & Kramer, M. (2015). The manifest dream report and clinical change. In Kramer, M. , & Glucksman, M. (Eds.), Dream research. Contributions to clinical practice (pp. 107–123). Routledge.

[joap70011-bib-0022] Greenberg, R. , & Pearlman, C. (1978). If Freud only knew. A reconsideration of psychoanalytic dream theory. International Review of Psycho‐Analysis 5, 71–75.

[joap70011-bib-0023] Hall, C. S. , & Van De Castle, R. L. (1966). The content analysis of dreams. Appleton‐Century‐Crofts.

[joap70011-bib-0024] Hartmann, E. (2010). The nature and functions of dreaming. Oxford University Press.

[joap70011-bib-0025] Hill, C. E. (1996). Working with dreams in psychotherapy. Guildford Press.

[joap70011-bib-0026] Hill, C. E. (2004). Dream work in therapy. Facilitating exploration, insight, and action. American Psychological Association. 10.1037/10624-000

[joap70011-bib-0027] Iftikhar, M. , Tahir, K. , Falak, S. , & Shabbir, N. (2020). Dreams and waking life connection. International Journal of Dream Research, 13(2), 220–228.

[joap70011-bib-0028] Jung, C. G. (1944). Psychologie und Alchemie. Rascher.

[joap70011-bib-0029] Jung, C. G. (1969). General aspects of dream psychology. CW 8.

[joap70011-bib-0030] Kächele, H. , Eberhardt, J. , & Leuzinger‐Bohleber, M . (1999). Expressed relationships, dream atmosphere and problem solving in Amalia’s dreams—Dream series as process tool to investigate cognitive changes—A single case study. Psychoanalytic Process Research Strategies, 2. Ulmer Textbank.

[joap70011-bib-0031] Kächele, H. , Leuzinger‐Bohleber, M. , Buchheim, A. , & Thomä, H. (2006). Amalie X—ein deutscher Musterfall (Ebene I und Ebene II). Psychoanalytische Therapie (pp. 121–174). Springer.

[joap70011-bib-0032] Kächele, H. (2012). Dreams as subject of psychoanalytical treatment research. In Fonagy, P. , Kächele, H. , Leuzinger‐Bohleber, M. , & Taylor, D. (Eds.), The significance of dreams. Bridging clinical and extraclinical research in psychoanalysis (pp. 89–100). Karnac.

[joap70011-bib-0033] Leuzinger‐Bohleber, M. (1989). Veränderung kognitiver Prozesse in Psychoanalyse, Fünf aggregierte Einzelfallstudien, 2, PSZ‐Drucke. Springer.

[joap70011-bib-0034] Leuzinger‐Bohleber, M. (2013). Embodiment—Traum(a)—Depression. In Janta, B. ; Unruh, B. , & Walz‐Pawlita, S. (Hg.), Der Traum. Psychosozial‐Verlag.

[joap70011-bib-0035] Levin, R. (1990). Psychoanalytic theories on the function of dreaming. A review of the empirical dream research. In Masling, J. M. (Ed.), Empirical studies of psychoanalytic theories. Erlbaum.

[joap70011-bib-0036] Mitscherlich, A. (1983). Gesammelte Schriften: Psychosomatik, Bd. 2. Suhrkamp.

[joap70011-bib-0037] Moser, U. , & von Zeppelin, I. (1996). Die Entwicklung des Affektsystems. Psyche, 50(1), 32–84. 8584745

[joap70011-bib-0038] OPD‐Task Force (2008). Operationalized psychodynamic diagnosis OPD‐2. Manual of diagnosis and treatment planning. Hogrefe.

[joap70011-bib-0039] Roesler, C. (2018a). Structural Dream Analysis: A narrative research method for investigating the meaning of dream series in analytical psychotherapies. International Journal of Dream Research, 11(1), 21–29.

[joap70011-bib-0040] Roesler, C. (2018b). Dream content corresponds with dreamer’s psychological problems and personality structure and with improvement in psychotherapy. A typology of dream patterns in dream series of patients in analytical psychotherapy. Dreaming, 28(4), 303–321.

[joap70011-bib-0041] Roesler, C. (2019). Jungian theory of dreaming and contemporary dream research—Findings from the research project “Structural Dream Analysis”. Journal of Analytical Psychology, 65(1), 44–62. 10.1111/1468-5922.12566 31972892

[joap70011-bib-0042] Roesler, C. (2023). Dream interpretation and empirical dream research—An overview of theoretical developments in psychoanalysis and empirical findings. The International Journal of Psychoanalysis *,* 104(2), 301–330. 37139735 10.1080/00207578.2023.2184268

[joap70011-bib-0043] Roesler, C. (forthcoming, 2025). Dreams and dream interpretation: A contemporary introduction. Routledge Introductions to Contemporary Psychoanalysis. Routledge.

[joap70011-bib-0044] Roesler, C. (in review, accepted). A re‐analysis of classic cases of psychoanalytic dream interpretation with Structural Dream Analysis. International Journal of Dream Research.

[joap70011-bib-0045] Roesler, C. , Kissling, L. , Sütterlin, T. , Gees, A. (2024). Dreams in psychotherapy: An empirically supported model of the relations of dreams to the course of psychotherapy. International Journal of Dream Research, 17(2), 164–176.

[joap70011-bib-0046] Roesler, C. , & Widmer, D. B. (2023). Amalia revisited—A reanalysis of Amalia’s dreams with the method Structural Dream Analysis. Brain Sciences, 13 *,* 796–807. 10.3390/brainsci13050796 37239268 PMC10216061

[joap70011-bib-0047] Sándor, P. , Szakadát, S. , & Bódizs, R. (2016). The development of cognitive and emotional processing as reflected in children’s dreams: Active self in an eventful dream signals better neuropsychological skills. Dreaming, 26(1), 58–78.

[joap70011-bib-0048] Schredl, M. (2018). Researching dreams. Palgrave McMillan.

[joap70011-bib-0049] Schredl, M. , Germann, L. , & Rauthmann, J. (2022). Recurrent dream themes: Frequency, emotional tone, and associated factors. Dreaming, 32(3), 235–248.

[joap70011-bib-0050] Varvin, S. , Fischmann, T. , Jovic, V. , Rosenbaum, B. , & Hau, S. (2012). Traumatic dreams: Symbolisation gone astray. In Fonagy, P. et al. (Eds.), The significance of dreams (pp. 182–211). Karnac.

[joap70011-bib-0051] Vömel, S. (2020). Ansatz zur Validierung der Strukturalen Traumanalyse als diagnostisches Inventar und Ableitung therapeutischer Interventionen zur Persönlichkeitsentwicklung. Unpublished Masterthesis, Diploma Fachhochschule Nordhessen.

[joap70011-bib-0052] Zadra, A. , & Domhoff, G. W. (2010). Dream content: Quantitative findings. In Kryger, H. M , Roth, T. , & Dement, W. C. (Eds.), Principles and practice of sleep medicine (pp. 585–594). Saunders/Elsevier.

